# The effect of *Chlorella* supplementation in pregnant women with low‐grade inflammation

**DOI:** 10.1002/fsn3.3759

**Published:** 2023-10-16

**Authors:** Yoko Uchiyama‐Tanaka, Fumi Shimabukuro, Eri Okumura, Masaki Fujishima

**Affiliations:** ^1^ Yoko Clinic Kitakyushu, Fukuoka Japan; ^2^ Yui Clinic Noborikawa Okinawa Japan; ^3^ Sun Chlorella Corp Kyoko Japan

**Keywords:** Chlorella, constipation, low‐grade inflammation, pregnant women

## Abstract

Pregnancy dramatically changes maternal metabolism and the microbiome. Low‐grade inflammation can cause maternal complications and fetal abnormalities. The objective of this open‐label, randomized, controlled study was to evaluate the efficacy and safety of orally administered *Chlorella*, a green alga that is commercially available as a dietary supplement with rich nutrients and dietary fiber for pregnant women with low‐grade inflammation. Patients with C‐reactive protein levels >0.05 mg/dL (16 weeks gestation, *n* = 22) were enrolled and randomly allocated to the *Chlorella* group (*n* = 10) or control group (*n* = 12). We conducted blood biochemical tests at 25, 30, and 35 weeks gestation and evaluated the evacuation status (symptoms depending on the Rome IV C2 criteria and laxative usage), side effects, and complications throughout the investigation. We also monitored the status of the offspring. The *Chlorella* group (*n* = 0) showed a significantly lower rate of constipation than the control group (*n* = 8). This study demonstrated the beneficial effects and safety of *Chlorella* supplementation in pregnant women, which prevented constipation and unnecessary laxative administration.

## Abstract 2

Chlorella dietary supplements contain essential amino acids and chlorophyll as well as minerals, such as iron, vitamins and fiber. Chlorella supplementation in pregnant patients with low‐grade inflammation was safe and decreased the prevalence of constipation.

## 1 INTRODUCTION

Pregnancy induces a major shift in hormones, metabolism, the immune response, and the microbiome (Amir et al., 2020). The prevalence of constipation during pregnancy is reported to range from 11% to 38%, with etiologies including rising progesterone levels, changes in fluid and fiber intake, changes in the gut microbiome, and supplementation, such as with iron (Amir et al., 2020; Vazquez, 2010). Low‐grade chronic inflammation in the gut, vagina, or oral cavity during pregnancy has been correlated with health problems in both pregnant women and fetuses (Bumrungpert et al., 2022; Choi et al., 2016; Kim et al., 2022; Yeates et al., 2020). Low‐grade inflammation increases the risk of labor complications, preterm birth, prematurity, autism, and other diseases of offspring in the future (Amir et al., 2020; Bumrungpert et al., 2022; Choi et al., 2016; Kim et al., 2022; Vazquez, 2010). The intake of healthy food is important for pregnant women, and a poor diet and/or the inability to process food for bioavailability reduces the body's coping ability and can create an environment that favors disease and metabolic disorders (Rook & Brunet, 2005; Yeates et al., 2020).

*Chlorella* is a unicellular green alga that grows in fresh water and contains much more protein and chlorophyll than other plants. *Chlorella* species are high in vitamins, including multiple forms of folate and vitamins B12 and D, minerals such as iron and magnesium, and dietary fiber (Marik, 2012; Mizoguchi et al., 2008; Nakano et al., 2010). *Chlorella pyrenoidosa* (*C. pyrenoidosa*) polysaccharides can modulate the gut microbiota by promoting beneficial bacteria, inhibiting the growth of harmful bacteria, and reducing the ratio of *Firmicutes* to *Bacteroides*. *C. pyrenoidosa* polysaccharides can increase short‐chain fatty acid content, which specifically interacts with the immune system, including a switch toward tolerogenic populations, thereby conferring maternal health benefits and favoring healthy immune imprinting in the foetus (Miyazawa et al., 2013; Nakano et al., 2007).

*Chlorella* species and *Chlorella* extracts have been reported to exert a variety of effects, including reducing inflammation and cholesterol levels, preventing stress‐induced ulcers, enhancing resistance to infection and antineoplastic activity, decreasing dioxins in breast milk, and reducing the risk of anemia, proteinuria, and edema in pregnant women. *Chlorella* is currently widely commercially available as a nutritional supplement and health food (Lv et al., 2022; Marik, 2012; Mizoguchi et al., 2008; Nakano et al., 2010; Van Der Linde et al., 2021).

In the present study, we assessed the efficacy of *Chlorella* supplementation for pregnant women with low‐grade inflammation.

## 2 METHODS

### 2.1 Population

The present study was conducted from March to July 2017, and the procedures were in accordance with the guidelines of the Declaration of Helsinki (2000) for human experimentation. Twenty‐two outpatients from Yui Clinic (Okinawa, Japan; age range, 23–38 years old) were enrolled in the current study. This study protocol was approved by the ethics committee of the Shiba Palace Clinic in Tokyo, Japan, and all participants gave informed consent.

### 2.2 Materials and study design

Subjects with a C‐reactive protein (CRP) level >0.05 mg/dL (16 weeks gestation, *n* = 22) were enrolled, and those with obvious infection or acute inflammation were excluded. Subjects were randomly allocated to the *Chlorella* group, which received oral *Chlorella* tablets (*C. pyrenoidosa*, Sun *Chlorella* Corp.) containing dried *Chlorella* (mean age 34.9 ± 3.5 years old, parity 2.8; *n* = 10), or the control group, which did not (mean age 31.6 ± 3.7 years old, parity 2.0; *n* = 12), according to the month they visited the clinic. The subjects in the *Chlorella* group were supplemented daily with 6 g of *Chlorella* supplement (30 tablets) from 12 to 18 weeks gestation until delivery. There was no limitation on the use of multivitamins and minerals.

We conducted blood biochemical tests to evaluate the red blood cell (RBC) count and hemoglobin (Hb), hematocrit (Ht), serum iron, ferritin, CRP, folate, vitamin B12, homocysteine, and albumin levels at the beginning of the study and at 25, 30, and 35 weeks gestation. All blood samples were drawn from the antecubital vein. We monitored constipation (symptoms depending on the Rome IV C2 criteria and laxative usage), iron intake (start criteria: Hb level < 11 g/dL), side effects, and other complications throughout the investigation. The status of each newborn (body weight, height, Apgar score) was also assessed.

### 2.3 Statistical analyses

Statistical analyses were performed on all endpoint parameters in the protocol group (*n* = 20 participants who completed the study). All parameter values across all timepoints were analyzed, and no data were excluded. The results are presented as the means ± SD. Data were collected at 16, 25, 30, and 35 weeks gestation. Data were analyzed via the R‐statistical software program, version 4.1.2 (R Project for Statistical Computing) using Fisher's exact tests, paired *t* tests, Student's *t* tests, and Dunnett's tests. Differences were considered significant at *p* < .05.

## 3 RESULTS

Table 1 shows the general clinical findings of the subjects. After random allocation, the age, parity, and CRP levels were (by chance) significantly higher in the *Chlorella* group than in the control group before the intervention. Two subjects in the control group were unable to complete blood sample collection (one because of mismanagement and one due to withdrawal from the study because of severe pregnancy‐induced hypertension [PIH] and eclampsia). The labor complications and newborn characteristics are shown in Table 2. There were no significant differences in the newborns' heights, weights, or Apgar scores between the two groups. Table 3 shows the results of blood biochemical tests performed at 16, 25, 30, and 35 weeks gestation. Although the CRP level at 16 weeks gestation was higher in the *Chlorella* group than in the control group, there were no significant differences at 35 weeks gestation. Hb and ferritin levels were controlled equally as needed based on the iron supply, but the dosages were significantly lower in the *Chlorella* group than in the control group at 35 weeks gestation. Six of the 10 patients in the control group and 2 of the 10 patients in the *Chlorella* group voluntarily took multivitamin minerals (Table 4). Both groups needed iron supplementation (6 of the 10 patients in the *Chlorella* group and 8 of the 12 patients in the control group). There was no significant difference in the rate of constipation between the two groups before the intervention; however, after the intervention, the *Chlorella* group had a significantly lower rate of constipation than the control group (*Chlorella* group: 0 of the 10 patients; control group: 8 of the 12 patients, with 5 needing laxatives; Table 5).

**TABLE 1:** Subject characteristics.

	*Chlorella* group	Control group	*p* value
	*N* = 10	*N* = 10	
Age (years)	34.9 ± 3.5	31.6 ± 3.7	.055
Height (cm)	156.2 ± 4.6	158.5 ± 5.2	.309
Weight (kg)	49.0 ± 4.6	50.9 ± 5.2	.383
Parity (number of births)	2.8 ± 1.0	2.0 ± 0.5	<.05
Preintervention CRP level (mg/dL)	0.21 ± 0.08	0.13 ± 0.06	<.05
Preintervention Hb level (g/dL)	12.2 ± 1.0	12.1 ± 0.8	.773

*Note*: Differences between groups were calculated using Student's *t* test.

**TABLE 2:** Days of delivery and newborn characteristics.

	*Chlorella* group	Control group	*p* value
	*N* = 10	*N* = 7–10	
Apgar score, first minute	8.3 ± 1.1	8.3 ± 0.5	.974
Apgar score, second minute	9.3 ± 0.5	8.9 ± 0.4	.061
Newborn height (cm)	49.6 ± 3.4	49.2 ± 1.8	.749
Newborn weight (g)	3023 ± 367	3095 ± 330	.651

*Note*: Differences between groups were calculated using Student's *t* test.

**TABLE 3:** Blood biochemical test results.

	CRP level		Hb level		Ferritin level	
	*Chlorella* group	Control group	*Chlorella* group	Control group	*Chlorella* group	Control group
Week	*N* = 10	*N* = 10	*N* = 10	*N* = 10	*N* = 10	*N* = 10
16	0.21 ± 0.08^†^	0.13 ± 0.06	12.2 ± 1.0	12.1 ± 0.8	14 ± 7	16 ± 14
25	0.24 ± 0.22	0.68 ± 1.73	11.0 ± 1.0*	11.0 ± 0.8**	7 ± 3**	5 ± 2*
30	0.21 ± 0.16	0.46 ± 0.90	11.3 ± 0.8	11.2 ± 0.6*	7 ± 3**	6 ± 2*
35	0.17 ± 0.09	0.22 ± 0.19	11.5 ± 0.6	11.3 ± 0.4*	9 ± 5*	7 ± 3*

*Note*: The means were compared using analysis of variance, followed by Student's *t* test. ^†^ *p* < .05 vs control group. The means were compared using analysis of variance, followed by Dunnett's test. *p <  .05, **p < .01 versus 16 week group.

**TABLE 4:** Use of laxatives and supplements during pregnancy.

	Chlorella group	Control group	*p* value
	*N* = 10	*N* = 12	
Laxatives	0 (0)	5 (41.7)	<.05
Iron supplements	6 (60.0)	8 (66.7)	1.000
Multivitamins	2 (20.0)	6 (50.0)	.204

*Note*: Data are presented as the number and (percentage). Fisher's exact test was performed.

**TABLE 5:** Constipation before and after the intervention.

	*Chlorella* group	Control group	*p* value
	*N* = 10	*N* = 12	
Before	6 (60.0)	5 (41.7)	.721
After	0 (0)	8 (66.7)	<.01

*Note*: Data are presented as the number (percentage). Fisher's exact test was performed.

## 4 DISCUSSION

The present findings suggest that the use of *Chlorella* supplementation during pregnancy can decrease constipation and the need to take laxatives. The prevalence of constipation is approximately 30% during pregnancy due to changes in the hormonal status, eating behaviors, and the microbiome (Amir et al., 2020; Brosseau et al., 2021; Vazquez, 2010). Parity and a history of cesarean section have been associated with constipation (Baldassarre et al., 2019; Hannah et al., 2004; Marshall et al., 1998). The use of prebiotics and probiotics during pregnancy reportedly induces beneficial effects (Saurel‐Cubizolles et al., 2000; Van Der Linde et al., 2021). Prebiotics are fermented by gut bacteria, leading to the release of short‐chain fatty acids (Van Der Linde et al., 2021). In patients with low‐grade inflammation, the frequency of these issues increases with the use of medicine and supplements. In addition, the frequent use of laxatives is also problematic not only for ensuring a safe pregnancy but also for maintaining good nutritional dynamics. In recent years, many studies have demonstrated the importance of the intestinal environment, including its relationship with diseases, the relationships among organs, and analyses of intestinal microbiota and metabolites.

At the start of pregnancy and during the first trimester, the gut microbial diversity in pregnant women appears to be similar to that in nonpregnant women; however, a substantial shift in phylogenetic composition and structure occurs over the course of pregnancy. From the first to third trimester, the proportion of Proteobacteria increased from 0.73% to 3.2%, and that of Actinobacteria increased from 5.1% to 9.3%. Phylogenetic diversity decreases in the third trimester. High levels of estrogen and progesterone during pregnancy have been reported to increase susceptibility to *Listeria monocytogenes* infection (Amir et al., 2020). Even low‐grade inflammation during pregnancy influences infant growth and is related to the development of autism, other mental disorders, and other medical conditions (Bumrungpert et al., 2022; Jewell & Young, 2000; Wawer et al., 2021).

Energy and micronutrient requirements in pregnant women are particularly high because of the increased maternal metabolic rate and fetal demands. Diet is very important for preventing constipation and microinflammation, but pregnant women often experience changes in their food preferences and immunity, making it difficult to consume the optimal volumes of fiber and fluids. A healthy diet and lifestyle involving stress management are important for pregnant women. A recent study suggested that the consumption of a mixed diet containing folate‐rich foods can be almost as effective as folic acid supplementation in improving folate status (Perichart‐Perera et al., 2017). We instructed subjects to improve their diet, but this proved to be difficult for many patients because of hyperemesis, busy lives (e.g., taking care of other children), mental status, and pregnancy‐induced changes in meal preferences.

*Chlorella* is a natural ingredient that includes dihydrofolates and tetrahydrofolates, such as 5‐10‐MTHF and 5‐MTHF. Compared with other plant supplements, *Chlorella* dietary supplements contain abundant essential amino acids and chlorophyll as well as large quantities of minerals, such as iron and magnesium, and vitamins, such as vitamins B6 and B12 (Lv et al., 2022). *Chlorella* is reported to reduce the oxidation of erythrocyte membrane lipids (Miyazawa et al., 2013), and dioxin reduces and improves anemia in pregnant women (Nakano et al., 2010). The mechanism underlying these influences is unclear at present, but rich dietary fiber or thiol residue chelates toxic substances, including dioxin and oxidative low‐density lipoproteins (LDLs) and heavy metals (Mizoguchi et al., 2008; Nakano et al., 2010). Beneficial nutrients, such as essential amino acids, multiform folate, and vitamins B6, B12, and D, facilitate metabolism and regeneration as well as DNA biosynthesis. *Chlorella* supplementation also depends on the individual gut environment (Winkels et al., 2007), so managing one's daily lifestyle and food intake to ensure a healthy gut environment before pregnancy is very important. In the present study, the control group took multivitamin minerals but still needed laxatives. There is a Developmental Origins of Health and Disease (DOHaD) concept that links an individual's health and risk of disease in later childhood and adulthood with their environmental upbringing, especially their diet, in early life (Nishimoto et al., 2021). *Chlorella* is rich in micronutrients, such as folate, vitamin B12, and fiber, which might also benefit fetal growth.

The safety of *Chlorella* supplementation has also been reported (Lv et al., 2022; Marik, 2012; Mizoguchi et al., 2008; Nakano et al., 2010). *C. pyrenoidosa* polysaccharides can promote the growth of *Parabacteroides distasonois* and increase the short‐chain fatty acid content, thereby contributing to the promotion of intestinal health and prevention of disease. *C. pyrenoidosa* polysaccharides have prebiotic functions with different fermentation characteristics from those of conventional prebiotics, such as fructooligosaccharides, and may thus represent new prebiotics for improving human health (Nakano et al., 2007).

Both groups in the present study were allowed to freely supplement their diets with multivitamin micronutrients. The main differences between the two groups were the incidence of constipation in the *Chlorella* group, which was related more to parity than to a greater tendency of constipation. This result suggests that *Chlorella* might be useful as a prebiotic beyond simple supplementation with micronutrients. There were several limitations in this study. There were not enough participants, so we could not identify significant differences in the decreased CRP levels and nutrient status of pregnant women. The random grouping method was based on the month of visiting the clinic, so we should correct for no differences between the two groups, such as age, preintervention CRP levels and parity. This was an open‐label study.

## 5 CONCLUSION

*Chlorella* supplementation in pregnant patients with low‐grade inflammation was safe and decreased the prevalence of constipation. We do not know the exact mechanism in this study. There were some limitations, such as the sample size and group planning. We would like to further analyze the changes in the microbiota of the *Chlorella* and control groups in the future. We hope to study this issue in more patients by performing a double‐blind randomized control study.

## AUTHOR CONTRIBUTIONS

**Yoko Uchiyama‐Tanaka:** Conceptualization (lead); data curation (lead); formal analysis (supporting); funding acquisition (equal); investigation (supporting); methodology (lead); project administration (lead); supervision (lead); visualization (equal); writing – original draft (lead); writing – review and editing (lead). **Fumi Shimabukuro:** Investigation (lead); resources (lead). **Eri Okumura:** Formal analysis (supporting); resources (supporting); software (supporting); validation (equal). **Masaki Fujishima:** Funding acquisition (equal); resources (equal); software (equal); validation (equal); writing – original draft (supporting).

## FUNDING INFORMATION

There is no funding for this research.

## CONFLICT OF INTEREST STATEMENT

The authors Y.U. and F.S. have no competing interests to declare that are relevant to the content of this article. E.O. and M.F. are employees of Sun Chlorella Corp.

## ETHICS STATEMENT

This study protocol was approved by the ethics committee of the Shiba Palace Clinic in Tokyo, Japan (UCHIR‐001), and all participants gave informed consent.

## INFORMED CONSENT

The procedures were in accordance with the guidelines of the Declaration of Helsinki (2000) for human experimentation. Informed written consent was obtained from all participants.

## Data Availability

The datasets generated and/or analyzed during the current study are available from the corresponding author on reasonable request.
